# Evaluation of arterial digital blood flow using Doppler ultrasonography in healthy dairy cows

**DOI:** 10.1186/s12917-017-1090-8

**Published:** 2017-06-06

**Authors:** H. Müller, M. Heinrich, N. Mielenz, S. Reese, A. Steiner, A. Starke

**Affiliations:** 1 Clinic for Ruminants and Swine, Faculty of Veterinary Medicine, University of Leipzig, An den Tierkliniken 11, 04103 Leipzig, Germany; 2 Institute for Agriculture and Nutrition, Biometrics and Informatics in Agriculture Group, Martin-Luther-University, Karl-Freiherr-von-Fritsch-Straße 4, 06120 Halle (Saale), Germany; 3 Institute of Anatomy, Histology, and Embryology, Department of Veterinary Sciences, Faculty of Veterinary Medicine, Ludwig-Maximilians-University, Veterinaerstraße 13, 80539 Munich, Germany; 4 Clinic for Ruminants, Vetsuisse-Faculty, University of Bern, Bremgartenstrasse 109a, 3012 Bern, Switzerland

**Keywords:** Arterial blood flow, Cattle, Foot, Pulsed wave Doppler, Ultrasonography

## Abstract

**Background:**

Local circulatory disturbances have been implicated in the development of foot disorders in cattle. The goals of this study were to evaluate the suitability of the interdigital artery in the pastern region in both hind limbs using pulsed-wave (PW) Doppler ultrasonography and to investigate quantitative arterial blood flow variables at that site in dairy cows. An Esaote MyLabOne ultrasound machine with a 10-MHz linear transducer was used to assess blood flow in the interdigital artery in the pastern region in both hind limbs of 22 healthy German Holstein cows. The cows originated from three commercial farms and were restrained in a standing hoof trimming chute without sedation.

**Results:**

A PW Doppler signal suitable for analysis was obtained in 17 of 22 cows. The blood flow profiles were categorised into four curve types, and the following quantitative variables were measured in three uniform cardiac cycles: vessel diameter, pulse rate, maximum systolic velocity, maximum diastolic velocity, end-diastolic velocity, reverse velocity, maximum time-averaged mean velocity, blood flow rate, resistance index and persistence index. The measurements did not differ among cows from the three farms. Maximum systolic velocity, vessel diameter and pulse rate did not differ but other variables differed significantly among blood flow profiles.

**Conclusions:**

Differences in weight-bearing are thought to be responsible for the normal variability of blood flow profiles in healthy cows. The scanning technique used in this report for evaluation of blood flow in the interdigital artery appears suitable for further investigations in healthy and in lame cows.

## Background

Diseases of the locomotor system are of major welfare and economic importance in dairy cattle. The prevalence of lameness can range from 21 to 55%, depending on the geographical region, farm management, and observer [1–4]. Nearly 90% of lameness is caused by diseases of the foot [5], which have a multifactorial aetiology; the structure of the stable flooring, the design of cubicles as well as standing and lying times all play a role [4, 6, 7]. Currently, the most common foot disorders in dairy cows are claw horn lesions associated with laminitis (sole ulcer and white line disease) and digital dermatitis [3, 8, 9].

Scanning electron microscopy studies have revealed vascular alterations in feet collected post mortem from cows with laminitis and digital dermatitis [10]. In-vitro studies have shown that ischaemia results in a reduction in oxygen and glucose transfer from the perioplic and coronary epidermis to keratinocytes of the bovine claw leading to reduced cell viability and abnormal cell differentiation [11]. It has been suggested that blood circulation plays an important role in the development of foot disorders in cattle [12]. Therefore, the effects of the type of barn flooring on digital blood flow should be investigated to better understand pathophysiological implications of vascular changes in the feet of dairy cows [13].

A variety of techniques have been used for assessing blood vessels in the bovine foot. Angiography was used to show that vascular changes play a role in the pathogenesis of laminitis in bovine feet collected post mortem [14, 15] and in the feet of anaesthetised cattle [16, 17]. Brightness (B)-mode ultrasonography is a reliable diagnostic tool for the identification of abnormalities in bones, tendons, ligaments and synovial structures in the bovine foot [18, 19] and has been used to examine blood vessels in the feet of healthy cows [20]. Pulsed-wave (PW) Doppler is a non-invasive technique for the evaluation of blood perfusion of an organ. Doppler ultrasonography has been used in cattle to study uterine blood flow [21–23], fatty liver syndrome [24], the musculophrenic vein [25] and as a tool for echocardiography [26]. A literature search (pubmed, October 2016, keywords: PW Doppler limb cattle) failed to identify publications describing the use of PW Doppler ultrasonography in the bovine foot. In contrast, Doppler ultrasonography has been used widely in horses for the examination of blood flow in the distal limb, although it is not yet a routine diagnostic procedure. Doppler ultrasonography has been used to monitor vascular blood flow dynamics in the feet of horses with laminitis and septic pododermatitis [27], to study the temporal relationship between lipopolysaccharide-induced decrease in digital blood flow and the appearance of vasoconstrictor mediators in plasma [28], to determine the repeatability of ultrasonographic measurements and the variability in blood flow in weight-bearing and non-weight-bearing limbs [29], and to determine the effects of acetylpromazine and xylazine on digital blood flow in standing horses [30].

The goal of this study was to evaluate the suitability of the interdigital artery in the pastern region in both hind limbs for PW Doppler ultrasonographic examination in cattle. A secondary goal was to investigate quantitative arterial blood flow variables relative to the blood flow profiles in the hind feet of healthy dairy cows.

## Methods

The study was approved under the guidelines of the Research Animal Act (research permit number 24–9168.21/4/30; A 30/12) by the Regional Council of Saxony, Department of Veterinary Public Health Service and Foodstuffs Control, Dresden, Germany.

### Animals and study design

Twenty-two healthy German Holstein cows (age 2.2 to 5.6 (mean 4.0) years, lactation number 1 to 3 (mean 2.3), days in milk 52 to 147 (mean 95), milk yield 36.2 ± 7.1 kg / day (mean ± standard deviation), body mass 652 ± 84 kg, withers height 143 ± 5 cm, body condition score 2.5 to 3.75 / 5 (mean 3.0)) were enrolled. The cows were from three commercial free-stall farms in Saxony, Germany (farm 1, *n* = 11; farm 2, *n* = 5; farm 3, *n* = 6). They underwent an on-farm clinical examination, which included lameness scoring [31], and all feet were trimmed [32] 1 day before ultrasonographic examination of both hind limbs. Blood samples were collected from the external jugular vein into heparinised and EDTA tubes for the determination of haematocrit and the concentrations of urea, creatinine and total protein. All measured blood variables were in the respective reference intervals [33, 34].

### Ultrasonographic examination

The unsedated cows were placed in a standing hoof-trimming chute and scanned by an experienced examiner (HM) using a MyLabOne ultrasound machine (Esaote Deutschland GmbH, Germany) with a linear transducer (linear array 33 mm; 10 MHz) without a standoff pad. The hair of both hind limbs was clipped from the tarsal region to the coronary band 1 day before the scan. Immediately before the scan, the clipped lower legs were washed with soap (Baktolin, Bode Chemie GmbH, Germany), warm water and a soft brush. Ultrasound gel (Henry Schein GmbH, Germany) was applied with a paintbrush, and both hind limbs, referred to as locations, were scanned in random order. The behaviour of the cow, leg position [31], duration of examination and the ambient temperature during the examination were recorded. An attempt was made to examine the cows when they were standing squarely, but forceful correction of the stance was not done to avoid undue excitement of the cows. The following scoring system was used for behaviour:

- 0 - calm - occasional movements of the hind limbs
- 1 - agitated - frequent movements of the hind limbs
- 2 - excited - frequent movements of the hind limbs accompanied by swishing of the tail and turning of the head toward the hind limbs.

**Fig. 1:**
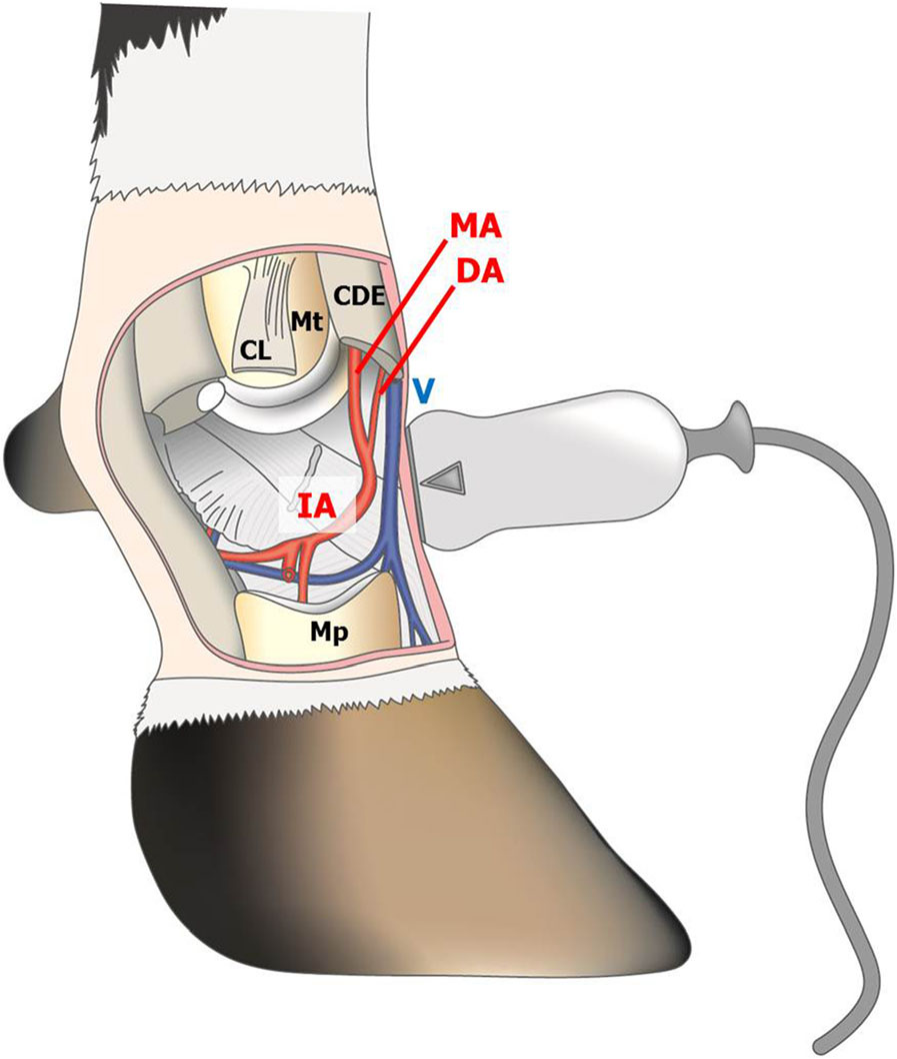
Schematic longitudinal view of a bovine foot showing the position of the linear transducer for pulsed-wave Doppler examination of the interdigital artery in the pastern region distal to the convergence of the dorsal metatarsal artery III with the dorsal common digital artery III. The digit closest to the viewer has been removed to show the plantar course of the artery between the digits. MA = dorsal metatarsal artery III, DA = dorsal common digital artery III, IA = interdigital artery, V = dorsal common digital vein III, CDE = common digital extensor tendon, CL = collateral ligament, Mt = metatarsal bone IV, Mp = middle phalanx

The ultrasonographic examination started with B-mode using a protocol described by Heppelmann et al. [35] for examination of the soft tissues, joints and bone surfaces, and a protocol by Kofler [20] for examination of the blood vessels. Pulsed-wave Doppler mode was then used to measure blood flow in a longitudinal section of the interdigital artery in the pastern region distal to the convergence of the dorsal metatarsal artery III with the dorsal common digital artery III [36] (Fig. 1). In this region, the interdigital artery runs from the dorsal aspect of the foot in a plantar direction between the digits. The suitability of the interdigital artery for PW Doppler ultrasonography was determined in a preliminary trial. The penetration depth was set at 3 cm. When the widest section of the artery was clearly visualised, beam steering (10°) was used to ensure that the angle between blood flow direction and ultrasound waves was less than 60°. The B-mode focus zone was set at the level of the vessel (1.25 or 1.75 cm image depth), and a low level of gain was used to obtain a slightly dark B-mode picture. Pulsed-wave ultrasonography in the duplex mode was then started while placing the transducer on the artery with as little pressure as possible. The Doppler volume sample cursor was placed in the middle of the vessel and occupied half to two-thirds of the vessel diameter. The Doppler angle was set in the vessel course. The pulse repetition frequency and level of the zero line were adapted to the blood flow velocity. The PW gain was adjusted to obtain optimum contrast between the Doppler wave form and the background [37]. Self-prepared pre-sets were used to minimise the time required to obtain an image of recordable quality. One image recording a PW Doppler signal of at least 5 s was stored for each measurement.

### Analysis of PW Doppler flow characteristics

The PW Doppler ultrasound images and data were transferred to the analysis program MyLabDesk (Esaote Biomedica Deutschland GmbH, Germany) on a laptop. Three similar cardiac cycles of the recorded measurement series were analysed by the same examiner (HM). The profile of the cardiac cycles was evaluated qualitatively and assigned to one of four types.

**Fig. 2:**
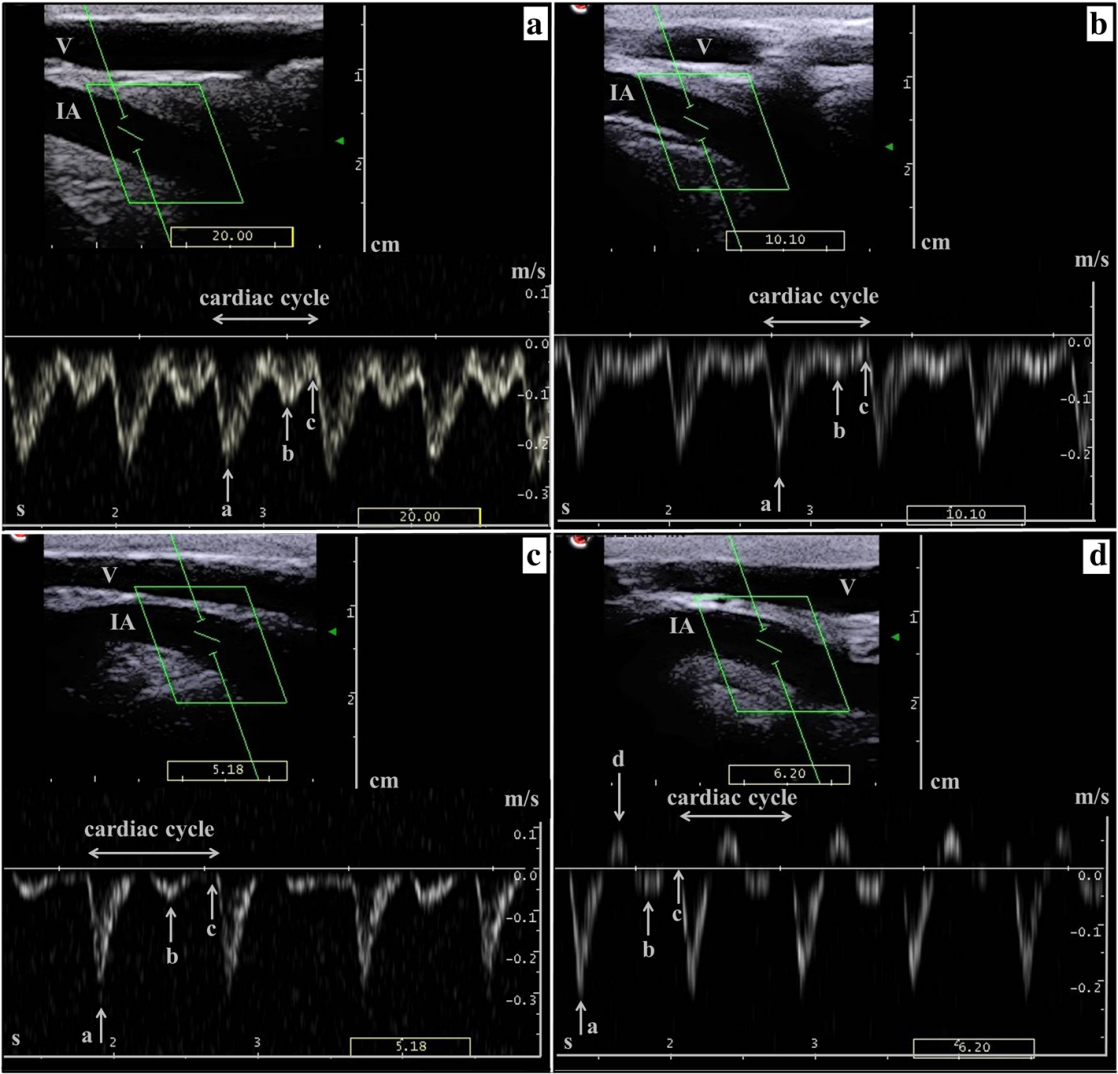
The four different blood flow profiles of the interdigital artery in the pastern region distal to the convergence of the dorsal metatarsal artery III with the dorsal common digital artery III. The image on top shows the interdigital artery in sagittal section and brightness mode. Below is the pulsed wave Doppler curve. **a** Type 1. The blood flow profile is biphasic without reverse blood flow, and the maximum diastolic velocity is at least half of the maximum systolic velocity. **b** Type 2. The blood flow profile is biphasic without reverse blood flow, and the maximum diastolic velocity is between one fourth and one third of the maximum systolic velocity. **c** Type 3. The blood flow profile is biphasic without reverse blood flow, and the blood flow velocity approaches the zero line between the maximum systolic and the maximum diastolic velocities. **d** Type 4. The blood flow profile is characterised by a triphasic waveform with one episode of reverse blood flow. IA = interdigital artery, V = dorsal common digital vein III, a = maximum systolic velocity (Vsyst), b = maximum diastolic velocity (Vdiast), c = end-diastolic velocity (Venddiast), d = reverse velocity (Vrev)

- Type 1 - *biphasic blood flow profile without reverse blood flow*; the maximum diastolic velocity (Vdiast) is at least half of the maximum systolic velocity (Vsyst; Fig. 2a)
- Type 2 - *biphasic blood flow profile without reverse blood flow*; the Vdiast is between one fourth and one third of Vsyst (Fig. 2b)
- Type 3 - *biphasic blood flow profile without reverse blood flow*; blood flow to the zero line between the Vsyst and Vdiast (Fig. 2c)
- Type 4 - *triphasic blood flow profile with one or two episodes of reverse diastolic blood flow* (Fig. 2d).

The following quantitative variables were measured at each location: vessel diameter (D), pulse rate (PR), maximum systolic velocity, maximum diastolic velocity, end-diastolic (Venddiast) and reverse velocity (Vrev). The maximum time-averaged mean velocity (Vmean = FVI × t; t = duration of one pulse cycle, FVI = flow velocity integral, FVI = ∑Vi × ΔT, ΔT = time interval, Vi = instant velocity), blood flow rate (BF = Vmean × π × (D / 2)^2^, resistance index (biphasic waveform RI = Vsyst – Venddiast / Vsyst or triphasic waveform RI = Vsyst – Vrev / Vsyst) and persistence index (biphasic waveform PI = Vsyst – Venddiast / Vmean or triphasic waveform PI = Vsyst – Vrev / Vmean) were calculated.

### Statistical analyses

The blood flow variables were statistically analysed using a linear model with correlated residual effects. The analysis was based on the following model with regard to farm and location:

(A) y_ijk_ = μ + α_i_ + β_k_ + (αβ)_ik_ + e_ijk_ with cov (e_ij1_, e_ij2_) = ρ × σ_e1_ × σ_e2_

where y_ijk_ is the response variable of the j-th animal from the i-th farm (*i* = 1,2,3) at the k-th location (k = 1,2), μ is an intercept, α_i_ is the fixed effect of the i-th farm, β_k_ is the fixed effect of k-th location, (αβ)_ik_ is the interaction between farm and location, and e_ijk_ is the random residual effect, which is assumed to have a normal distribution. The term “location” refers to left or right hind limb. A non-significant interaction term was dropped from the model. The residual effects of the locations on the same animal were assumed to be dependent with correlation ρ and with heterogeneous residual variances. The parameter ρ can be interpreted as the phenotypic correlation between the observations of the two locations on the same animal.

Phenotypic correlations between different traits were estimated by using two-trait analyses within locations. Because of the analysis within the locations, the linear models contain only the fixed effects of the farms and random residual effects.

Because of the small sample size, simultaneous analysis of the factors farm, location and curve type in one complex model was omitted. With model A, most variables did not differ significantly among farms and between locations and therefore an alternative model B was used for the analysis of the curve types:

(B) y_ijk_ = μ + α_i_ + e_ijk_ with cov (e_ij1_, e_ij2_) = ρ × σ_e1_ × σ_e2_

where y_ijk_ is the response variable of the j-th animal with the i-th curve type (*i* = 1,2,3,4) at the k-th location (k = 1,2), μ is an intercept, α_i_ is the fixed effect of the i-th curve type and e_ijk_ is the random residual effect, which is assumed to have a normal distribution. As with model A, correlations of residual effects were considered because of repeated measurements within cows.

The MIXED procedure (SAS, version 9.4, SAS Institute Inc., Cary, N.C., USA) was used for estimation of the model parameters. Differences between means were analysed using the F-test or the Tukey-Kramer test with Kenward-Roger approximation to calculate the degrees of freedom. The Shapiro-Wilk test was used to examine the studentized residuals for normality. Results were presented as least square means. Differences were considered significant at *p* < 0.05.

## Results and discussion

### Measurability of blood flow in the interdigital artery and selection of the measuring point

A PW Doppler signal suitable for analysis could not be obtained in either of the hind limbs in five of the 22 cows. In these ten hind limbs, the following B-mode conditions accompanied the lack of a suitable PW Doppler signal:

- good B-mode image of the artery, no PW Doppler signal (*n* = 2)
- moderate B-mode image of the artery, no PW Doppler signal (*n* = 5)
- moderate B-mode image of the artery, PW Doppler signal obtained but not suitable for analysis (*n* = 3).

The lateral palmar digital artery at the level of the fetlock joint [38–40] and the medial palmar digital artery [27] have been used for PW Doppler ultrasonography in horses. The plantar common digital arteries II, III and IV have a diameter of only 1 to 2 mm in cattle and cannot be imaged ultrasonographically in all cows [20]. By contrast, the dorsal metatarsal artery III has a diameter of 4 mm [20] and can be consistently visualised ultrasonographically at the level of the fetlock joint in cattle. Visualisation of this artery further distally (interdigital artery) is difficult because the vessel courses between the digits toward the plantar aspect of the foot [20]. Therefore we had hoped to identify a suitable measuring site for the dorsal metatarsal artery III at the level of the metatarsus in preliminary trials. PW Doppler ultrasonography of dorsal metatarsal artery III in the metatarsal region was only possible when a make-shift standoff pad was used to obtain a Doppler angle smaller than 60°; suitable reusable diagonal standoff pads for the linear transducer that we used were not commercially available. To achieve a Doppler angle smaller than 60°, we selected the measuring site in a longitudinal section of the interdigital artery in the pastern region distal to the convergence of the dorsal metatarsal artery III with the dorsal common digital artery III. The less-than-ideal visualisation of the artery by B-mode ultrasonography in this region and the low occlusive pressure of the transducer (in an attempt to minimise compression of the artery) were possible reasons for the lack of suitable Doppler signals in five cows.

### Ambient temperature during ultrasonography

The ambient temperature ranged from 4 to 24 °C (mean 11 °C) and the examination room was draught-free. It was not possible to maintain a constant ambient temperature for all cows, similar to studies in horses [40, 41]. However, the barn and the examination room had a common air space and therefore the cows did not encounter major changes in ambient temperature when moved from the barn to the examination room.

### Behaviour and leg position during ultrasonography

Thirteen of the 22 cows were calm, three were agitated and six were excited during the examination. Several attempts were necessary to obtain a PW Doppler signal in agitated and excited cows. A PW Doppler signal suitable for analysis could not be obtained in three calm and two agitated cows. There were no significant associations between behaviour and quality of B-mode images and behaviour and the generation of PW Doppler images suitable for analysis.

The position of the hind limbs of the 17 cows from which a useful PW Doppler signal was obtained was normal (*n* = 20 hind limbs) or slightly forward (*n* = 14 hind limbs). To maintain normal blood circulation, the limbs were not fixed, and forceful manipulation of the leg position was avoided. Although the administration of xylazine did not change blood flow velocity or the diameter of the musculophrenic vein in cattle [25], it reduced the blood flow rate in the toe of healthy horses [30]. Therefore, we did not sedate the cows and instead dealt with a certain amount of shifting of weight between the limbs.

### Results of the B-mode ultrasonography

There were no abnormal findings on B-mode ultrasonograms of the larger blood vessels, synovial structures, tendons, bone surfaces, and skin.

### Results of the PW Doppler ultrasonography

#### Qualitative results - blood flow profiles

The distribution of the curve types of the different blood flow profiles is shown in Table 1. Six cows had the same curve type in both locations. Curve type 4 was only seen in images of the right hind limb. There were no significant associations between the order of measurement and curve type and the duration of examination and curve type.

**Table 1:** Distribution of four types of blood flow profiles seen on PW Doppler ultrasound images of the interdigital artery in the pastern region of the left and right hind limb in 17 healthy German Holstein cows

Curve type	Left hind limb	Right hind limb	Total
Type 1	3	2	5
Type 2	11	7	18
Type 3	3	3	6
Type 4	0	5	5

Biphasic as well as triphasic arterial blood flow profiles have been described in humans; the former occurs with low peripheral resistance in an artery [37]. Diastolic blood flow decreases with increasing peripheral vascular resistance as for example with an increased perfusion demand or ischaemia [37]. In humans, triphasic blood flow profiles are typical of peripheral arteries and they occur when peripheral resistance is high. This flow pattern can change to a biphasic pattern when vasodilatory second messengers are released, e.g. physical activity [37]. Healthy horses had biphasic blood flow profiles with one systolic peak, two or three diastolic peaks and an end-diastolic plateau [39–41]. Biphasic blood flow profiles also occur in horses with laminitis, pododermatitis [27, 40] and systemic inflammatory response syndrome [40]. Triphasic blood flow profiles have been documented in healthy horses [27, 42] and in horses with laminitis [40]. Furthermore, the end-diastolic plateau can approach the zero line on PW Doppler ultrasound images from healthy horses when the examined limb bears more weight [29]. Blood flow profiles and various quantitative PW Doppler variables obtained from restless healthy horses had a higher variability than those obtained from relaxed healthy horses [29]. Degree of weight-bearing and blood flow rate in the limb were inversely related in healthy horses because weight-bearing increases peripheral resistance in the toe [29, 30] and causes the diastolic plateau to be lower. Therefore non-weight bearing limbs of healthy horses showed a low or non-resistance blood flow profile and weight bearing limbs a high resistance blood flow profile [29, 39]. The blood flow profile is prone to change particularly in diastole. We suspect that the different curve types observed in the present study were caused by variability in weight-bearing.

#### Quantitative results – Doppler parameters

The quantitative PW Doppler variables for both limbs are shown in Table 2 according to the statistical model A. The differences in LSMeans of the variable Vsyst (Δ 0.03 m / s), Vmean (Δ 0.02 m / s), and RI (Δ 0.11) between the hind limbs found in this study were significant but the amount seemed to be small. The diameter, Vdiast, Venddiast, BF, PR and PI did not differ significantly between locations. Quantitative PW Doppler variables measured at the various farms did not differ.

**Table 2:** Least square means (LSM) and standard error (SE) of the brightness mode and pulsed-wave Doppler variables of the interdigital artery in the pastern region in both hind limbs in 17 healthy German Holstein cows using statistical model A, which considered farm and location

Variable	*n*	LSM ± SE HL	LSM ± SE HR	p location	p farm
D (cm)	17	0.54 ± 0.02	0.54 ± 0.02	1.00	0.08
Vsyst (m/s)	17	0.35 ± 0.02	0.32 ± 0.02	0.04*	0.18
Vdiast (m/s)	17	0.12 ± 0.01	0.09 ± 0.01	0.06	0.35
Venddiast (m/s)	17	0.05 ± 0.01	0.03 ± 0.01	0.34	0.82
Vrev (m/s)	5		0.10 ± 0.02		
Vmean (m/s)	17	0.13 ± 0.01	0.11 ± 0.01	0.05*	0.63
PI^a^	16	2.18 ± 1.08	3.12 ± 1.38	0.10	0.09
RI	17	0.84 ± 0.02	0.95 ± 0.06	0.03*	0.24
BF^a^ (ml/min)	17	177.40 ± 1.10	131.10 ± 1.20	0.14	0.31
PR (1/min)	17	77 ± 2	77 ± 3	0.84	0.06

*D* vessel diameter, *Vsyst* maximum systolic velocity, *Vdiast* maximum diastolic velocity, *Venddiast* end-diastolic velocity, *Vrev* reverse velocity, *Vmean* maximum time-averaged mean velocity, *PI* persistence Index, *RI* resistance Index, *BF* blood flow rate, *PR* pulse frequency, *HR* right hind limb, *HL* left hind limb, *p location* differences between LSM of right and left hind limb, *p farm* F-test of differences between farms

*significance (*p* < 0.05)

^a^median in the original scale, calculated from the LSM estimated in the log transformed scale

The diameter of the interdigital artery was in general agreement with a mean diameter of 4 mm obtained at a similar measuring site further proximally in mature Simmental and Brown Swiss cows aged 6.1 ± 1.7 years and weighing 510 – 637 kg. The same study did not find a difference in vessel diameter between the limbs [20]. Horses with acute laminitis or pododermatitis (4.3 mm) had a larger diameter of the medial palmar digital artery than healthy horses (4.0 mm) [27]. The blood flow velocities and the resistance and persistence indices found in this study are largely comparable to the values measured in healthy horses [27, 39]. Blood flow velocities obtained from healthy horses were greater when the leg was lifted than when it was weight-bearing, but the resistance index did not differ [29]. In another study of healthy horses, diastolic and mean velocities decreased and resistance index increased with an increase in weight-bearing, whereas the maximum systolic velocity did not differ between weight-bearing and non-weight-bearing legs [39]. A possible reason for the discrepancy between these two studies with respect to maximum systolic velocity of weight-bearing and non-weight-bearing limbs could be a difference in the measuring site; in one study [39], the site was closer to the hoof capsule, where the effect of weight-bearing was more easily recognized, than in the other study [29]. The measuring point in our study was also close to the claw capsule. There was a remarkable difference in the Vsyst compared with that found by Pietra et al. [39]. However the difference in the mean of Vsyst between weight bearing (0.32 m / s) and non-weight bearing limbs (0.37 m / s) [29] were small. The difference in the diastolic velocities in healthy horses between weight bearing and non-weight bearing had a range from 0.03 to 0.1 m / s [29] or 0.02 to 0.04 m / s [39]. Also the RI had a similar range (0.09 [29], 0.07 [39]) like in this study. The described blood flow profiles in this two horse studies were consistent in the group of non-weight bearing and weight bearing limbs. Thus the comparison of the quantitative PW Doppler variables was between non- or low resistance and high resistance blood flow profiles. In our study the distribution of blood flow profiles differed considerably between locations, which could account for the difference in Vsyst, Vmean and RI. Horses with septic pododermatitis showed much higher amounts in blood flow velocities and BF than healthy horses [27] (e.g. Vsyst (mean) 0.52 vs. 0.31 m / s, Vmean 0.15 vs. 0.08 m / s, BF 90 ml / min vs. 40 ml / min).

The PR measured in the right and left hind limbs did not differ and the two rates were strongly correlated (*r* = 0.9, *p* = 0.001). This means that the degree of agitation or excitement did not change markedly between the examinations at the two locations.

In the left hind limb, Vdiast was positively correlated with Venddiast, Vmean and BF, and in the right hind limb, Vdiast was negatively correlated with the same variables (Table 3). Furthermore, the correlations between Vsyst and RI and between Vsyst and PI were stronger in the right than in the left hind limb. We suspect that these correlations depend on the distribution of the blood flow profiles between the hind limbs; types 3 and 4 are more resistive than types 1 and 2 and were more common in the right limb than in the left. Correlations between Vdiast and Venddiast, between Vdiast and Vmean, RI, PI and BF, and between Vendiast and Vmean, RI, PI and BF were similar for the two locations. The maximum systolic velocities measured at the two locations were positively correlated and Vdiast, Venddiast, Vmean and BF were negatively correlated. Even though the distribution of blood flow profiles differed between locations, maximum systolic velocities within cows were similar. In contrast, diastolic velocities varied considerably between locations. The hind limbs were not scanned simultaneously and therefore changes in the overall circulation or local factors could account for the lack of correlation between locations. However, major circulatory changes are unlikely because pulse rate and vessel diameter did not differ between locations, and therefore, local factors (weight-bearing, temperature) are more likely to be responsible.

**Table 3:** Correlations of pulsed-wave Doppler variables of both hind limbs and between hind limbs in 17 healthy German Holstein cows using statistical model A, which considered farm and location

		HL						
		Vsyst	Venddiast	Vdiast	Vmean	PI	RI	BF
**HR**	**Vsyst**	**0.77** ^c^	0.19^b^	0.44^b^	**0.67** ^b^	0.26^b^	0.25^b^	0.42^b^
	**Venddiast**	**−0.45** ^a^	−0.29^c^	**0.9** ^b^	**0.74** ^b^	**−0.79** ^b^	**−0.87** ^b^	**0.67** ^b^
	**Vdiast**	−0.28^a^	**0.75** ^a^	−0.26^c^	**0.86** ^b^	**−0.66** ^b^	**−0.67** ^b^	**0.80** ^b^
	**Vmean**	−0.27^a^	**0.79** ^a^	**0.87** ^a^	−0.45^c^	**−0.52** ^b^	−0.43^b^	**0.84** ^b^
	**PI**	**0.72** ^a^	**−0.69** ^a^	**−0.76** ^a^	**−0.79** ^a^	0.09^c^	**0.91** ^b^	**−0.55** ^b^
	**RI**	**0.64** ^a^	**−0.86** ^a^	**−0.61** ^a^	**−0.73** ^a^	**0.82** ^a^	0.27^c^	−0.41^b^
	**BF**	−0.37^a^	**0.60** ^a^	**0.82** ^a^	**0.89** ^a^	**−0.87** ^a^	**−0.59** ^a^	−0.26^c^

*Vsyst* maximum systolic velocity, *Vdiast* maximum diastolic velocity, *Venddiast* end-diastolic velocity, *Vmean* maximum time-averaged mean velocity, *PI* log transformed persistence index, *RI* resistance index, *BF* log transformed blood flow rate, *HR* right hind limb, *HL* left hind limb

bold text = different from zero (*p* < 0.05)

^a^correlations between pulsed-wave Doppler variables of the right hind limb

^b^correlations between pulsed-wave Doppler variables of the left hind limb

^c^correlations of the pulsed-wave Doppler variables between right and left hind limb

Therefore quantitative PW Doppler variables were also evaluated according to the statistical model B, where the blood flow profile was included without consideration of location and farm (Table 4). Vessel diameter, PR and Vsyst did not differ significantly among curve types but the remaining variables did in spite of the small sample size in curve type 3 and 4. Maximum systolic velocity, Venddiast, Vmean and BF decreased progressively from curve type 1 to curve type 4. The differences in LSMeans in the diastolic velocities (Vdiast Δ 0.1 m / s, Venddiast Δ 0.08 m / s) as well as in the Vmean (Δ 0.11 m / s) had a higher range between the curve types than in the Vsyst (Δ 0.05 m / s). Also the BF (Δ 200 ml / min) and the RI (Δ 0.55) differed strongly between the curve types.

**Table 4:** Least square means (LSM) and standard error (SE) of the brightness mode and pulsed-wave Doppler variables of the interdigital artery in the pastern region in both hind limbs in 17 healthy German Holstein cows using statistical model B, which considered curve type (CT)

Variable	LSM ± SE CT1	LSM ± SE CT2	LSM ± SE CT3	LSM ± SE CT4
D (cm)	0.56^a^ ± 0.03	0.55^a^ ± 0.02	0.51^a^ ± 0.03	0.55^a^ ± 0.03
Vsyst (m/s)	0.36^a^ ± 0.03	0.31^a^ ± 0.02	0.35^a^ ± 0.03	0.32^a^ ± 0.04
Vdiast (m/s)	0.16^a^ ± 0.01	0.10^b^ ± 0.01	0.09^b^ ± 0.01	0.06^b^ ± 0.01
Venddiast (m/s)	0.08^a^ ± 0.01	0.05^b^ ± 0.00	0.03^b^ ± 0.01	0.00^c^ ± 0.01
Vmean (m/s)	0.17^a^ ± 0.02	0.12^ab^ ± 0.01	0.11^bc^ ± 0.02	0.06^c^ ± 0.02
PI^e^	1.41^a^ ± 1.15	1.98^ab^ ± 1.08	3.04^b^ ± 1.15	13.38^c^ ± 1.37
RI	0.74^a^ ± 0.03	0.83^b^ ± 0.01	0.91^c^ ± 0.03	1.29^d^ ± 0.03
BF^e^ (ml/min)	267.86^a^ ± 1.21	164.23^a^ ± 1.10	134.02^ab^ ± 1.20	63.05^b^ ± 1.38
PR (1/min)	78^a^ ± 3	77^a^ ± 2	76^a^ ± 3	75^a^ ± 3

*D* vessel diameter, *Vsyst* maximum systolic velocity, *Vdiast* maximum diastolic velocity, *Venddiast* end-diastolic velocity, *Vmean* maximum time-averaged mean velocity, *PI* persistence index, *RI* resistance index, *BF* blood flow rate, *PR* pulse frequency

^a,b,c,d^ within rows, means with different superscripts are different (*p* < 0.05)

^e^median in the original scale, calculated from the LSM estimated in the log transformed scale

These findings support the view that a blood flow profile is primarily defined by diastole and Vsyst is independent of the curve type in healthy cows. The RI increased from type 1 to type 4, reflecting a parallel increase in peripheral resistance possibly related to an increase in weight-bearing as described before. Horses with triphasic blood flow profiles also had higher peripheral vascular resistance, which was negatively correlated with blood flow rate [43].

## Conclusions

It is possible to measure blood flow in the hind limbs of healthy dairy cows by using PW-Doppler ultrasonography. There appears a considerable variability of blood flow profiles, possibly related to shifting of weight during the measurements. The differences in the quantitative PW Doppler variables are of higher importance in the comparison of the curve types than in the comparison of the hind limbs despite of the small sample size. The blood flow velocities measured in this study are similar to velocities obtained at the lower legs of healthy horses. The technique and measuring site in the interdigital artery is well suited for further studies on the effects of different degrees of weight-bearing on blood circulation and development of claw disorders.

## Abbreviations

a: Maximum systolic velocity
b: Maximum diastolic velocity
BF: Blood flow rate
B-mode: Brightness mode
c: End-diastolic velocity
CDE: Common digital extensor tendon
CL: Collateral ligament
CT: Curve type
d: Reverse velocity
D: Vessel diameter
DA: Dorsal common digital artery III
EDTA: Ethylenediaminetetraacetic acid
FVI: Flow velocity integral
HL: Left hind limb
HR: Right hind limb
IA: Interdigital artery
LSM: Least square means
MA: Dorsal metatarsal artery III
Mp: Middle phalanx
Mt.: Metatarsal bone IV
PI: Persistence index
PR: Pulse rate
PW: Pulsed-wave
RI: Resistance index
SD: Standard deviation
SE: Standard error
t: Duration of one pulse cycle
V: Dorsal common digital vein III
Vdiast: Maximum diastolic velocity
Venddiast: End-diastolic velocity
Vi: Instant velocity
Vmean: Maximum time-averaged mean velocity
Vrev: Reverse velocity
Vsyst: Maximum systolic velocity
Δ: Difference
ΔT: Time interval
